# Shoulder Dysfunction After Radiotherapy in Surgically and Nonsurgically Treated Necks: A Prospective Study

**DOI:** 10.1097/MD.0000000000001229

**Published:** 2015-07-31

**Authors:** Qiang Sun, Shu Guo, Di Wang, Nan Xu

**Affiliations:** From the Department of Plastic Surgery, The First Affiliated Hospital of China Medical University, Shenyang, P.R. China.

## Abstract

Our goal was to evaluate the shoulder dysfunction after radiotherapy in surgically and nonsurgically treated necks.

A prospective pair matched design was performed. A total of 96 patients from 3 groups were enrolled in the study. The patients were asked to complete the shoulder domain section of the University of Washington Quality of Life questionnaire on 2 occasions: preoperatively and 12 months postoperatively.

None of the patients had a shoulder impairment before the operation. At the follow-up session, 4 patients who had received radiotherapy only reported mild shoulder dysfunction, the mean score was 96.3, the difference was significant compared with the preoperative score (*P* = 0.046). For patients who had received neck dissection, 7 patients reported that the impaired shoulder function caused them to change their work and 14 patients reported that their shoulder function was affected a little; the mean score was 71.6. For patients who had received both neck dissection and postoperative radiotherapy, 9 patients reported that they had changed their work due to shoulder dysfunction and 16 patients reported mild shoulder impairment; the mean score was 65.3 and the difference was not significant (*P* = 0.304).

Radiotherapy does not increase shoulder dysfunction in surgically treated necks, but it could induce shoulder impairment in nonsurgically treated necks.

## INTRODUCTION

Regional lymph node metastasis is one of the most important prognostic factors in head and neck cancer.^1,2^ Neck dissection, including selective neck dissection (SND) and modified radical neck dissection (MRND), has served as a valuable tool in treating neck diseases and adjuvant radiotherapy is often used for increasing regional control. Shoulder dysfunction is well documented after accessory nerve sparing neck dissection,^3,4^ its possible causes include direct neural injury, local devascularization, and decompression injury. However, the exact effect of postoperative radiation therapy on shoulder function remains unclear. Kuntz and Weymuller^3^ reported that the shoulder domain score from the University of Washington Quality of Life (UW-QoL) questionnaire did not show a significant correlation with radiotherapy treatment in patients; however, Schuller et al^4^ reported that postoperative radiation therapy could be detrimental to shoulder function. Therefore, in this study, we aimed to evaluate how radiotherapy could affect shoulder function in patients with head and neck cancers.

## PATIENTS AND METHODS

The China Medical University institutional research committee approved our study and all participants signed an informed consent agreement.

From January 2006 to December 2012, a total of 411 patients were diagnosed with head and neck cancer at the Department of Plastic Surgery, China Medical University. Of these patients, 34 patients underwent radiotherapy only, 123 patients underwent primary tumor resection and neck dissection, and 93 patients underwent primary tumor resection, neck dissection, and radiotherapy. All patients were asked to complete the shoulder domain section in the UW-QoL questionnaire preoperatively and 12 months postoperatively. To account for differences in certain factors such as age (±5 years), sex, tumor site, and similar, a matched-pair selection was performed in the 3 groups. Finally, 96 patients were enrolled (Table 1).

**TABLE 1:**
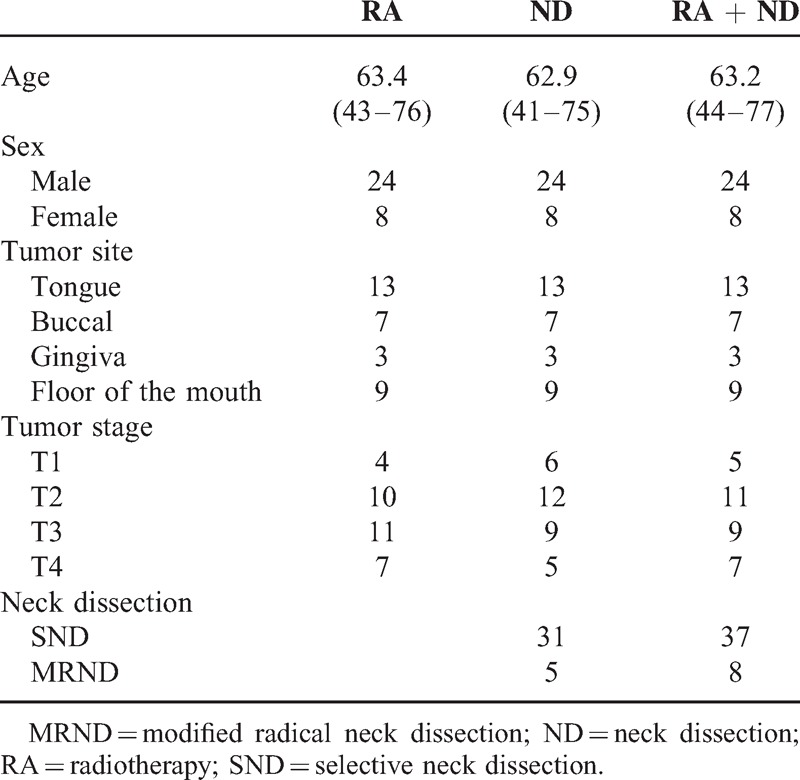
General Information of the Patients

The shoulder domain in the UW-QoL questionnaire consists of 4 responses and its scoring is scaled so that a higher score indicates better function; the 4 possible responses are scored as 0, 30, 70, and 100.

The Chi-squared test was used to assess the general variables. The nonparametric Mann–Whitney test was used to evaluate the UW-QoL scores, and all statistical analyses were performed using SPSS, Chicago, IL, USA 13.0. A *P* < 0.05 was considered significant.

## RESULTS

As described in Table 1, in each group (24 male and 8 female), there were 13 patients diagnosed with tongue carcinoma, 7 patients diagnosed with buccal carcinoma, 3 patients diagnosed with gingiva carcinoma, and 9 patients diagnosed with carcinoma of the floor of the mouth. In the patient group who had received neck dissection surgery, there were 31 SNDs and 5 MRNDs. In the patient group who had received neck dissection and postoperative radiotherapy, there were 37 SNDs and 8 MRNDs; the difference was not significant (*P* = 0.636).

As described in Table 2, none of the patients had the shoulder impairment before the operation. After leaving the hospital, 4 patients who had received radiotherapy only reported mild shoulder dysfunction; the mean score was 96.3, the difference was significant compared with the preoperative score (*P* = 0.046). Seven patients who had received neck dissection surgery reported impaired shoulder function that caused them to change the way they work and 14 patients reported that their shoulder function was affected a little; the mean score was 71.6. Nine patients who had received both neck dissection and postoperative radiotherapy reported that they had changed the way they work due to shoulder dysfunction and 16 patients reported a mild shoulder impairment; the mean score was 65.3, the difference was not significant (*P* = 0.304).

**TABLE 2:**
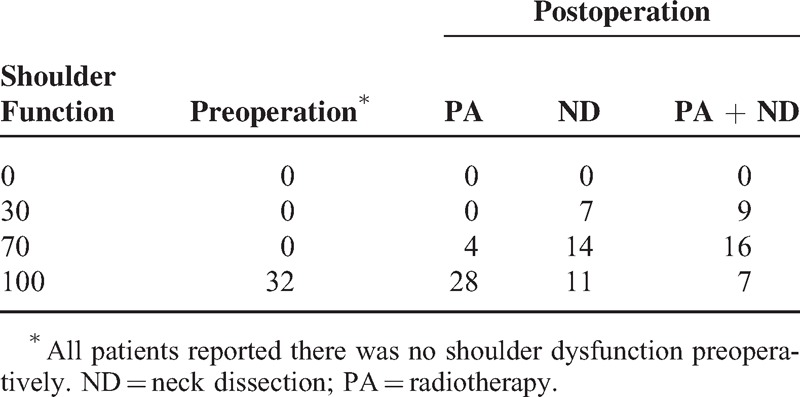
Shoulder Function in Patients

## DISCUSSION

Patients now pay more attention to postoperative complications after different therapeutic options as quality of life is a key consideration. Therefore, it was important in this study to evaluate the dysfunction from the patient's perspective. A number of methods are currently available for assessing shoulder function,^5,6^ but we chose the UW-QoL shoulder domain. Although this is an extremely simple measure and is limited to 1 of 4 responses, previous reports have proven its high reliability and sensitivity in screening for shoulder dysfunction.^7^

The exact effect of neck dissection on shoulder function has been well established. Guldiken et al^8^ reported that the preoperative stiffness and pain scores of patients undergoing SND were significantly better than those reported at the final visit using the Neck Dissection Impairment Index questionnaire. A study by Speksnijder et al^9^ is consistent with this, concluding that neck dissection is associated with deterioration of shoulder function. Furthermore, SND would have a better outcome, theoretically, if the nodes from level 5 were not included, in this case, the accessory nerve is at risk of injury, including neural injury, local devascularization, and decompression injury. Cappiello et al^10^ noted less shoulder dysfunction in patients undergoing levels 2 to 4 dissections compared with those receiving levels 2 to 5 dissections. A similar result was also reported by other authors^11^; MRND would indicate more “shoulder syndrome.” In this study, we noted that the mean postoperative score was 71.6 in patients with neck dissection, which was significantly lower than the preoperative score (*P* < 0.001); this was consistent with previous outcomes.

As to how radiotherapy affects shoulder function remains unclear. Karaman et al^12^ encountered shoulder syndrome by comparing the spinal accessory nerve latency and amplitude, and the authors found that latency levels of the spinal accessory nerve in patients without postoperative radiotherapy was significantly shorter than those undergoing adjuvant radiation treatment. Furthermore, Nowak et al^13^ reported that postoperative radiotherapy induced a 20% reduction in motion range of the shoulder. However, Watkins et al^5^ reported no significant difference in the subjective and objective Constant's scores between SND only versus SND plus radiotherapy. Erisen et al^6^ observed the effect of adjuvant radiotherapy on shoulder function by evaluating postoperative shoulder joint range of motion and electromyography, and the authors concluded that radiotherapy would not cause shoulder dysfunction. Similarly, Guldiken et al^8^ found that neither objective nor subjective measurements correlated with radiation therapy. All of the above-mentioned studies had their own shortcomings, such as imperfect design and small sample sizes. In our study, a prospective matched-pair design was performed which could account for the interference of some confounding factors. For example, previous reports have stated that age and tumor site had a significant association with postoperative shoulder function.^9,14^ We also noted that postoperative radiotherapy did not increase shoulder dysfunction. However, in our center, physical exercise is usually suggested for patients with neck dissection and physiotherapy also helps to promote shoulder function.^15^

Most previous studies focused on assessing the effect of radiotherapy on shoulder function in patients with neck dissection, but how about the effect on patients who had received nonsurgical treatment of the neck? Limited literature is available, however, van Wouwe et al^16^ reported that shoulder impairment did arise in patients who had received nonsurgical neck treatment after radiotherapy, but the extent was limited. However, the authors did not report whether the difference was significant compared with preoperation or volunteers. In this study, we also noted that 4 (12.5%) patients who had received radiotherapy only complained of mild shoulder dysfunction: this is consistent with previous findings, although the variation was significant compared with the preoperative score, the mean postoperative score was still as high as 96.3. Therefore, radiotherapy induces shoulder impairment in nonsurgically treated necks, but the extent was limited and well tolerated by the patients.

In summary, radiotherapy does not increase shoulder dysfunction in surgically treated necks, but it could induce shoulder impairment in nonsurgically treated necks, though the extent is limited.
