# Shoulder Dysfunction After Radiotherapy in Surgically and Nonsurgically Treated Necks: A Prospective Study: Erratum

**DOI:** 10.1097/01.md.0000475891.43472.db

**Published:** 2016-01-08

In the article “Shoulder Dysfunction After Radiotherapy in Surgically and Nonsurgically Treated Necks: A Prospective Study”,^1^ which appeared in Volume 94, Issue 30 of *Medicine*, Dr. Qi-gen Fang's name and affiliation were omitted from the original article. The article has since been corrected online.

## References

[1] Sun Q Guo S Wang D Shoulder dysfunction after radiotherapy in surgically and nonsurgically treated necks: A prospective study. *Medicine* 2015 94:e1229. 26222857 10.1097/MD.0000000000001229 PMC4554123

